# A novel 3D in vitro model of glioblastoma reveals resistance to temozolomide which was potentiated by hypoxia

**DOI:** 10.1007/s11060-019-03107-0

**Published:** 2019-01-29

**Authors:** Ahmed Musah-Eroje, Sue Watson

**Affiliations:** 1 Division of Cancer and Stem Cells, Cancer Biology, University of Nottingham, Nottingham, UK; 2 School of Life Sciences, University of Bedfordshire, Luton, UK

**Keywords:** Glioblastoma, 3D, Chemoresistance, Temozolomide, Hypoxia, CD133, OCT4

## Abstract

**Purpose:**

Glioblastoma (GBM) is the most common invasive malignant brain tumour in adults. It is traditionally investigated in vitro by culturing cells as a monolayer (2D culture) or as neurospheres (clusters enriched in cancer stem cells) but neither system accurately reflects the complexity of the three-dimensional (3D) chemoresistant microenvironment of GBM.

**Materials and methods:**

Using three GBM cell-lines (U87, U251, and SNB19), the effect of culturing cells in a Cultrex-based basement membrane extract (BME) [3D Tumour Growth Assay (TGA)] on morphology, gene expression, metabolism, and temozolomide chemoresistance was investigated.

**Results:**

Cells were easily harvested from the 3D model and cultured as a monolayer (2D) and neurospheres. Indeed, the SNB19 cells formed neurospheres only after they were first cultured in the 3D model. The expression of CD133 and OCT4 was upregulated in the neurosphere and 3D assays respectively. Compared with cells cultured in the 2D model, cells were more resistant to temozolomide in the 3D model and this resistance was potentiated by hypoxia.

**Conclusion:**

Taken together, these results suggest that micro-environmental factors influence GBM sensitivity to temozolomide. Knowledge of the mechanisms involved in temozolomide resistance in this 3D model might lead to the identification of new strategies that enable the more effective use of the current standard of care agents.

**Electronic supplementary material:**

The online version of this article (10.1007/s11060-019-03107-0) contains supplementary material, which is available to authorized users.

## Introduction

Glioblastoma is a devastating primary brain tumour. It is heterogeneous in nature and has a distinct cell of origin which makes it challenging to manage [1]. Temozolomide is the standard-of-care chemotherapy for glioblastoma [2]. However, most patients with glioblastoma die of the disease in less than 1 year after diagnosis as a result of chemoresistance associated with a cancer stem cell population and the topographically diffuse nature of the tumour [3]. To complicate this challenge, preclinical models that faithfully recapitulate relevant aspects of GBM biology in vitro have still not been established.

Tumour cells, including glioblastoma, are traditionally cultured in vitro on a plastic substrate, with an oxygen tension of 20%. However, the oxygen tension in GBM in vivo ranges from 0.1 to 10% [4]. Many studies do not take into account the physiologically relevant oxygen tension [5] as well as the changes in the extracellular matrix which can affect biological properties like proliferation and motility when investigating GBM cell in vitro [6].

Pre-clinical testing of drugs has largely relied on either two-dimensional (2D) in vitro cell models or animal studies. However, data from the 2D cultures are largely unreliable, as cells cultured in this model are not a true representative of the in vivo tumour microenvironment and, animal studies, as a result of interspecies differences, fail to fully recapitulate the response to drugs in humans with about 95% of anti-cancer drug candidates failing to make it to the clinic, thereby wasting significant time and resources [7]. The neurosphere assay is an acceptable 3D model for maintaining glioblastoma in vitro [8–10]. Although this assay has undoubtedly been regarded as the gold standard assay that selects for stem cell populations, it has a major drawback of allowing cells to form their own niche, with more differentiated cells positioned at the center than on the surface as well as containing a mixed population of cells and a small number of true stem cells [11].

3D cultures are now required as cells cultured in the 2D monolayers display aberrant cell–cell interactions [12]. As such, most of the conclusions from this system of culture do not accurately apply to the tumours in vivo. Therefore, 3D in vitro culture systems offer more realistic cell–cell and cell–matrix interactions that are more physiological providing a better alternative to the 2D systems [13, 14].

Recently, a 3D model based on polystyrene scaffold has been used to predict drug-radiation combination for glioblastoma [15]. Furthermore, glioblastoma tumour-initiating cells have been successfully maintained in a microscale alginate hydrogel tubes (or AlgTubes) that allows affordable cost for drug discovery [16].

To develop a 3D in vitro culture system for glioblastoma which will allow routine drug testing and molecular manipulation, e.g. gene knockdown, glioblastoma cell lines were established in a Cultrex-based 3-Dimensional Tumour Growth Assay (3D-TGA) that allows single cells to be extracted for further culturing. With this system, it was also possible to culture cells in a hypoxic tumour microenvironment (TME). The 2D and 3D models were compared to describe morphology, glioblastoma stem cell markers, metabolism as well as temozolomide chemosensitivity.

Taken together, the current results suggest that micro-environmental factors influence GBM cell biology. The 3D assay could be used to further characterize GBM cells, including potential stem cells, and the pathways through which the tumour microenvironment influences their characteristics and numbers, including their drug resistance and ability to invade/ metastasize.

## Materials and methods

### Cell-lines

U87 cells, from European Type Culture Collection (ECCC) while U251 and SNB19 cells from National Cancer Institute, NCI60, were grown in normoxic (20% oxygen) or hypoxic (1% oxygen) conditions as standard 2D culture and as a Cultrex-based 3D culture.

### The 3D-TGA

This was performed as previously described [14]. Briefly, the Cultrex basement membrane extract (BME) (Trevigen) was diluted to a concentration of 3 mg/ml on ice using phenol red-free modified RPMI (Life Technologies). The cells were resuspended at appropriate seeding density into a black-walled, low-adherent, clear-bottom 96-well plates (BrandTech) prewarmed to 37 °C.

## Neurosphere culture

Neurosphere cultures were maintained in 128 ml High Glucose Dulbecco’s Modified Eagle’s Medium (Invitrogen, UK) in which 116 ml F12 Ham (Invitrogen, UK) was added and supplemented by 10 ml B27 supplement (Invitrogen, UK), 100 µg/ml FGF (Invitrogen, UK) and 100 µg/ml EGF (Invitrogen, UK) as well as 100 mg/ml Heparin (Sigma-Aldrich), at 37 °C in a 5% CO_2_ and humidified atmosphere. Cells were seeded in a 24-well plate at a density of 20,000 cells/well.

### The culture of cells in a hypoxia chamber

The Invivo2 400 hypoxia workstation (Ruskinn Technology LTD) was used to set oxygen concentration at 1%. The chamber was accessed through an Ezee sleeve and purged with vacuum and gas pedal. The chamber which is set at 5% CO_2_ at 37 °C is attached to a nitrogen cylinder which helps to maintain oxygen concentration in the chamber.

### Quantitative real-time PCR

Gene expression was assessed using real-time RT-PCR and data expressed relative to the housekeeping gene, HPRT as previously reported [14]. The expression of CD133 and OCT4, which are commonly used to define the CSC-like population in brain tumours, were detected using SYBR Green (Eurogentec) and calculated using the ${2^{ - \Delta \Delta {{\text{C}}_{\text{t}}}}}$ method. The primer sequences used were: CD133 forward: 5′-CAATCTCCCTGTTGGTGATTTG-3′ and CD133 reverse: 5′-ATCACCAGGTAAGAACCCGGA-3′; OCT4 forward: 5′-GTTGGAGAAGGTGGAACCAA-3′ and OCT4 reverse: 5′-CTCCTTCTGCAGGGCTTTC-3′.

### Drug sensitivity assays

Temozolomide was dissolved in DMSO to a final concentration of 100 mM. Various concentrations ranging from 5 to 1500 µM was applied to cells in triplicate wells. The cells were exposed to the drugs for 3 days before final endpoint reading using the Alamar Blue assay. The Alamar Blue assay [Invitrogen; 10% (v/v), 37 °C for 1 h] was used both as an indicator of metabolic function and drug sensitivity using a fluorescent plate reader (Flex-Station II, Molecular Devices, CA, USA). Drug sensitivity was calculated as a percentage of matched untreated control and IC_50_ curves were plotted and values determined using GraphPad Prism 6 (GraphPad Software Inc., USA; nonlinear curve fit of *Y* = 100/(1 + 10^(LogIC50−X)^ × hillslope).

#### Statistics

Students-test from GraphPad Prism, version 6 was used to analyze all data. Data were analyzed with either t-test or one-way ANOVA (Turkey’s multiple comparison test).

## Results

### GBM cultured in the 3D model was viable and could be re-cultured as 2D monolayers or neurospheres

It has previously been demonstrated that the 3D model allows the culture of glioblastoma cells [14]. To know if cells cultured in the 3D can be subcultured, U251, U87 and SNB19 cells maintained in the 2D and 3D models were harvested and cultured as neurospheres.

**Fig. 1:**
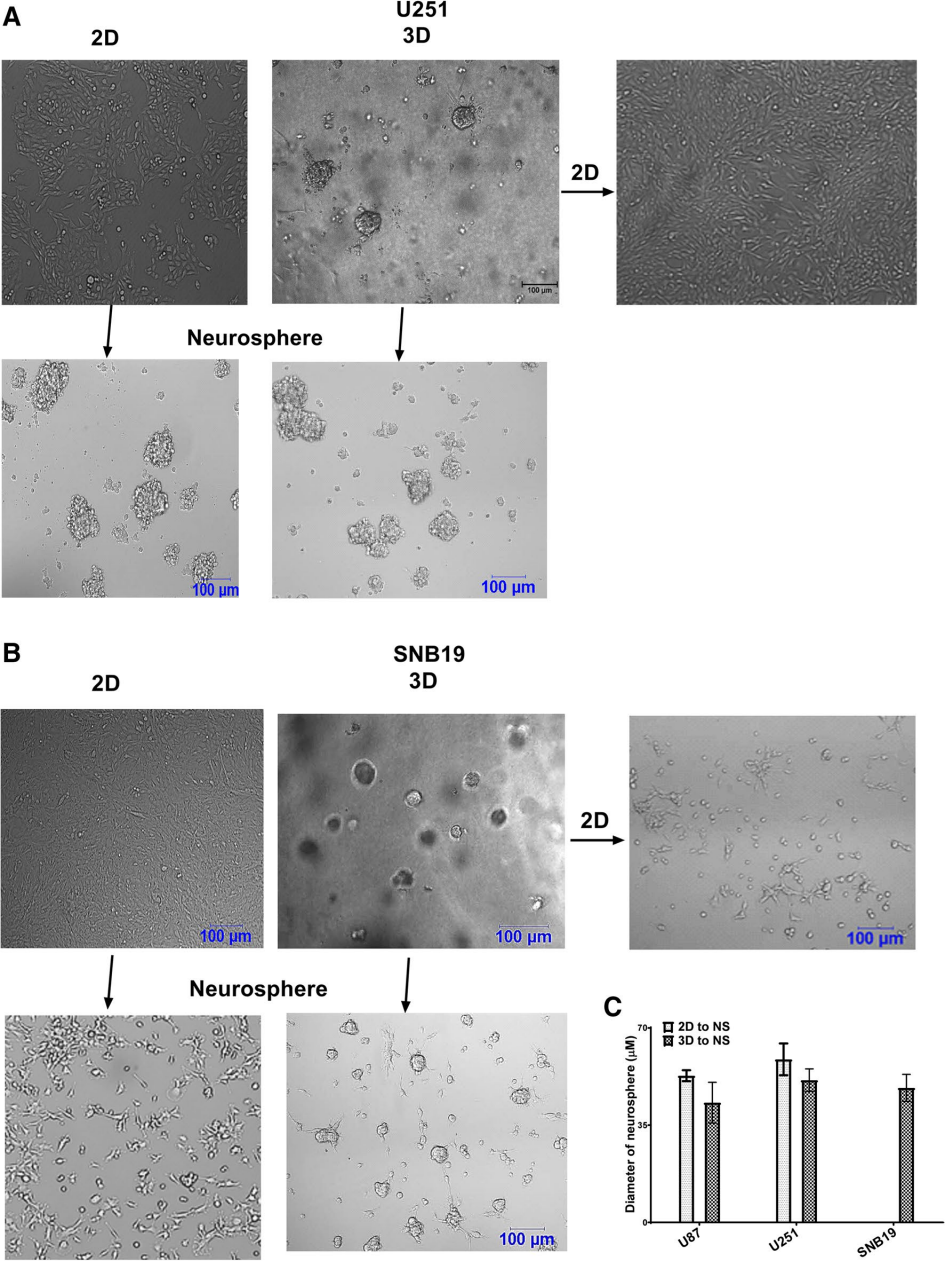
GBM cells presents distinct morphological feature in 3D and viable cells can be harvested from 3D and re-cultured as 2D and neurospheres. U251 (**a**) and SNB19 (**b**) cells were cultured in 2D and 3D models. The cells were harvested from these models and recultured as neurospheres.N = 3. Additionally, cells from the 3D models were recultured as 2D. **c** The diameter of neurospheres formed when cells were first cultured in the 2D and 3D model and recultured as neurospheres. Pictures were taken with a T9 Nikon Microscope. Scale bar = 100 µm. Magnification × 10

It was noted that only U251 (Fig. 1a) and U87 (Supp Fig. 1) cells formed neurospheres from the 2D model. Upon harvest from the 3D model, U251 cells formed a monolayer of cells and neurospheres (Fig. 1a). These neurospheres could be maintained for more than 6th generation (data not shown). Notably, the SNB19 cells did not form neurospheres after being maintained as a 2D monolayer (Fig. 1b). However, following culture in the 3D model, they formed neurospheres as well as monolayers of cells (Fig. 1b) indicating that culturing in the 3D model could influence the stem cell population to form neurospheres. Although the diameter of neurospheres formed when cells were first cultured in the 3D model was smaller than those formed when the cells were first cultured in the 2D model, this was not significant (Fig. 1c).

### Expression of stem cell markers, CD133 and OCT4 in the 2D-, 3D- and neurosphere assays

**Fig. 2:**
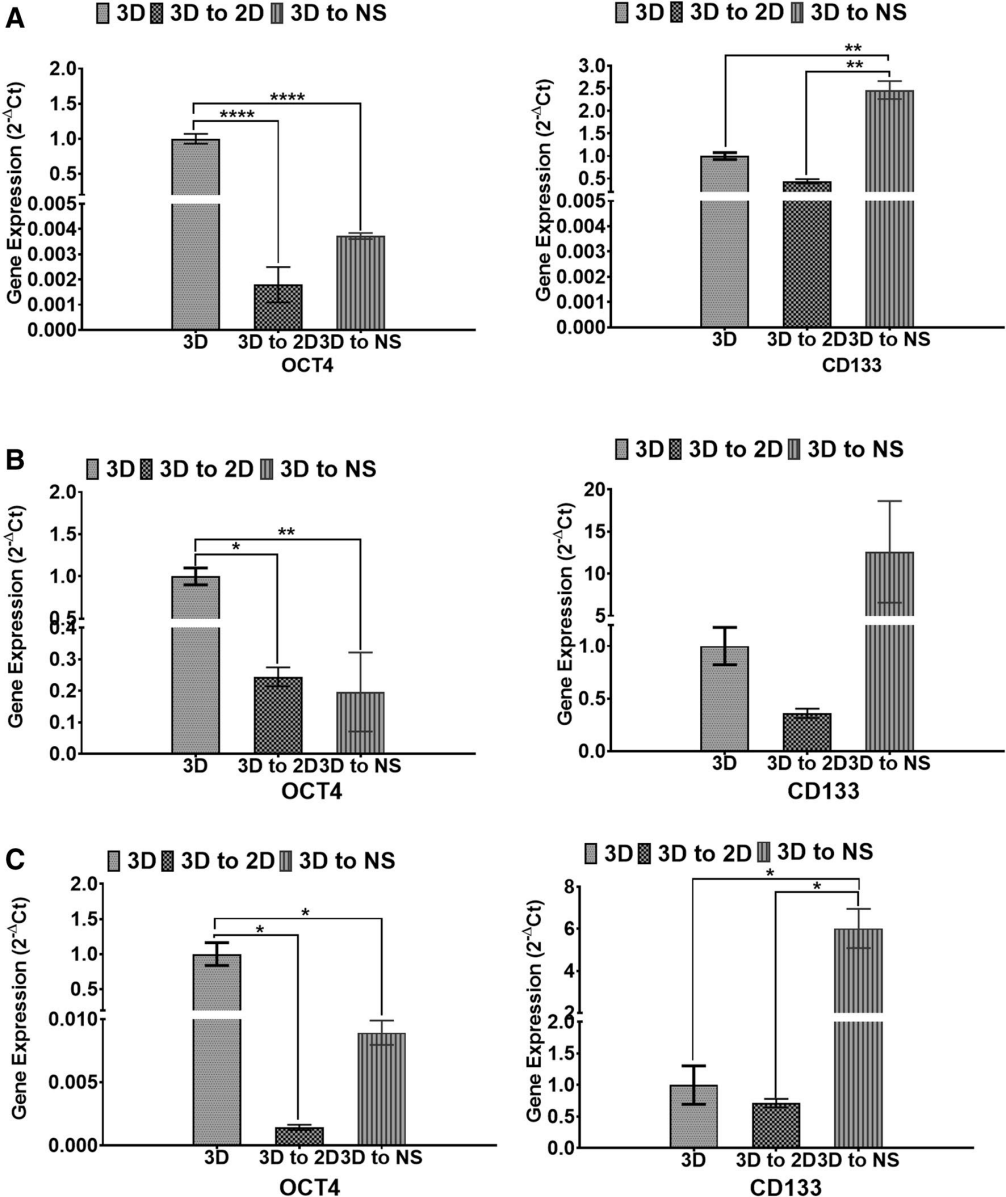
Differences in the mRNA expression of CD133 and OCT4 in the 2D, 3D and neurosphere assays. U251 (**a** and **b**), U87 (**c** and **d**) and SNB19 (**e** and **f**) cells were cultured as 3D. At day 3, the cells were harvested from the 3D matrix and recultured as either 2D (3D to 2D) or as neurospheres (3D to NS). qRT-PCR was used to quantify the levels of respective genes at day 3 (3D and 3D to 2D) and day 7 (3D to NS). The error bar represents standard error of mean from 3 independent experiments. One way ANOVA from Prism7 was used for statistical comparison. *p < 0.05, **p < 0.01, ****p < 0.0001. *NS* neurosphere

To determine the effect of the microenvironment on the expression of glioblastoma stem cell markers, we examined the mRNA expression of CD133 and OCT4 in the cells cultured in the 3D assay and cells cultured as 2D (3D to 2D) and neurospheres (3D to neurosphere) following extraction from the 3D assay (Fig. 1). Our result showed that CD133 expression was higher in the neurosphere assay compared to 2D and 3D assays with a significant upregulation in the U251 and SNB19 cells (Fig. 2; Table 1). Surprisingly, OCT4 mRNA expression was significantly upregulated in the cells cultured in the 3D assay compared to when the cells were recultured as 2D or neurospheres (Fig. 2; Table 1). This result suggests that the microenvironment plays an important role in the expression of different stem cell markers. It also indicates that different culture conditions can modulate the expression glioblastoma stem cell population.

**Table 1:** Fold difference of CD133 and OCT4 mRNA expression

	CD133		
	3D to 2D	2D versus NS	3D to NS
Fold difference			
U251	2.3 (NS)	5.6 (p = 0.0078)	2.5 (p = 0.003)
U87	2.8 (NS)	34.9 (NS)	12.6 (NS)
SNB19	1.4 (NS)	8.4 (p = 0.0167)	6.0 (p = 0.0142)

	OCT4		
	3D to 2D	2D versus NS	3D to NS
Fold difference			
U251	556.9 (p < 0.0001)	2.1 (NS)	268.6 (p < 0.0001)
U87	4.1 (p = 0.0123)	1.2 (NS)	5.1 (p = 0.0099)
SNB19	700.2 (p = 0.01)	6.3 (NS)	112.0 (p = 0.0103)

Following the culture of U251, U87 and SNB19 cells in the 3D assay, the cells were harvested from the 3D matrix and recultured as either 2D (3D to 2D) or as neurospheres (3D to NS). The fold difference relative to cells cultured in the 3D assay (3D to 2D and 3D to NS) is as indicated. In addition, the expression of genes in neurosphere NS assay relative to the 3D cells recultured as 2D is as indicated (2D vs. NS). The *p* values are as shown in brackets from One way ANOVA from Prism7. N = 3. *NS* not significant

### Metabolism pattern differs in the 3D model when compared with cells cultured in 2D in normoxia and hypoxia

**Fig. 3:**
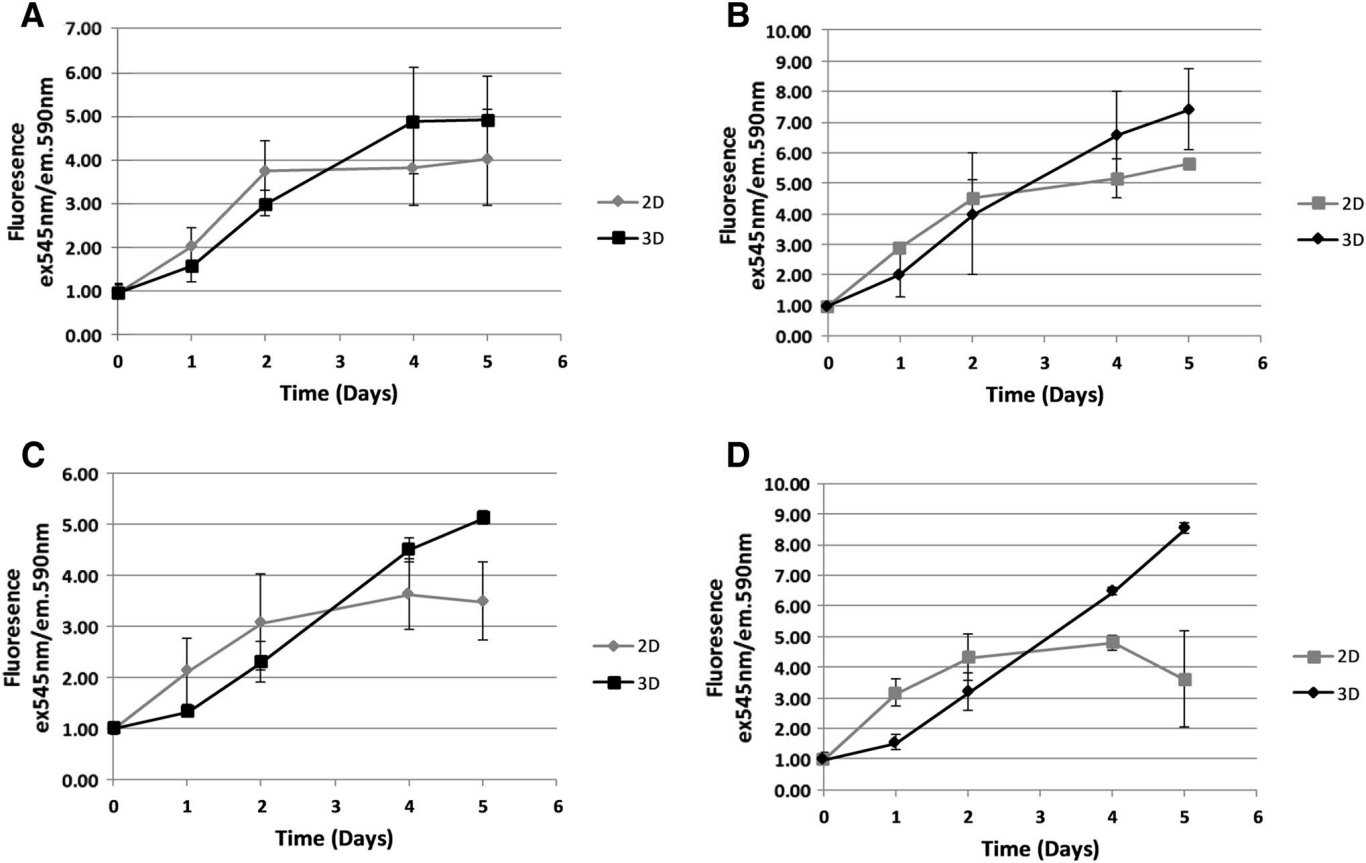
Metabolic activity of cells in the 2D and 3D assays in normoxia and hypoxia: U251 (**a** and **b**) cells and SNB19 cells (**c** and **d**) were cultured in the 2D (grey) and 3D (black) assays. At day 0 of set up, baseline reading was taken with the Alamar Blue assay after the cells had settled and one set of the cells was maintained in normoxia (left panel) while the other group was transferred to hypoxia (right panel). The metabolic activity of the cells was monitored for 5 days. The error bars represent the average fluorescence from 2 independent experiments. The graph was plotted relative to day 0

After establishing that GBM cells were viable in the 3D model and that they can be recultured, it was important to understand the influence of culture in the 3D model on metabolism as metabolism affects chemosensitivity. To achieve this, U251 and SNB19 cells were cultured in 2D and 3D in normoxia or hypoxia. The metabolic pattern as observed with the AlamarBlue assay in the 2D and 3D models was remarkable. After 2 days in the 2D model, metabolic activity from the readout was stabilized (Fig. 3a–c) and gradually decreasing in the SNB19 cells cultured in hypoxia (Fig. 3d). However, in the 3D model, a reduced metabolic readout was observed which gradually increased (Fig. 3a–d), with the U251 cells cultured in normoxia displaying constant reading between day 4 and 5 (Fig. 3a). In the U87 cells, metabolic activity was stabilised at day 3 in 2D assay but gradually increased from day 3 in the 3D assay (Supp Fig. 2). Attempt to understand the protein kinetics via western blot was technically difficult because of the time it took to harvest cells from the 3D matrix [14].

### The 3D confers resistance to temozolomide (TMZ) which was potentiated in hypoxia

**Fig. 4:**
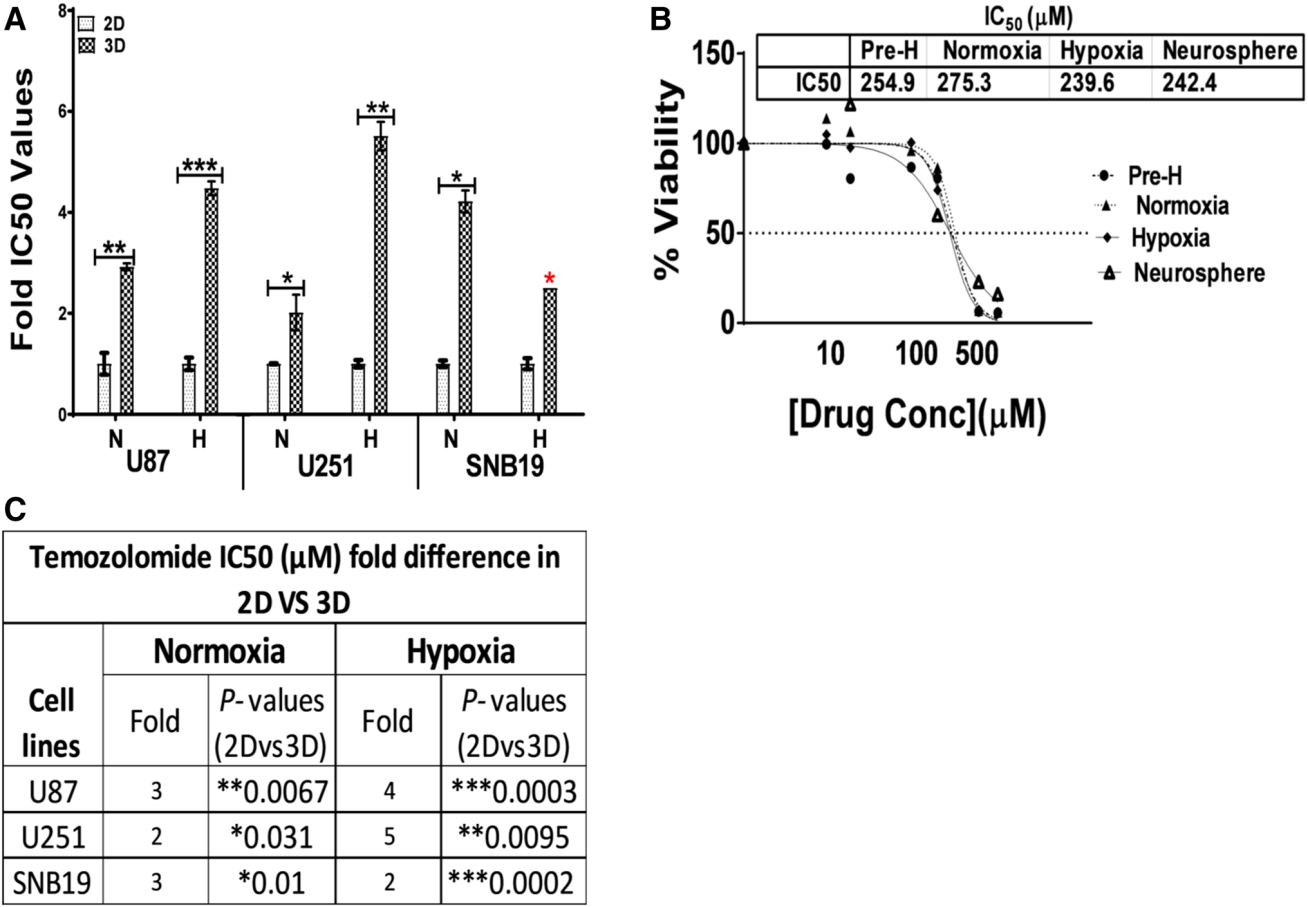
Sensitivity of GBM cells to temozolomide in the 2D, 3D and neurosphere assays in normoxia and hypoxia. **a** Following the culture of U87, U251 and SNB19 cells in the 2D and 3D models under normoxic (N) and hypoxic (H) conditions, the cells were treated with temozolomide and final viability was determined by AlamarBlue assay while GraphPad prism6 software was used to calculate IC_50_ values. The error bar represents standard error of the mean (SEM) from an average of 3 independent experiments. T-test from GraphPad prism6 was used for comparison. *Indicates that IC_50_ was not achieved and highest concentration was used. **b** U251 cells were cultured in the 2D model in three sets. At day 0 of setup, two sets were transferred to hypoxia while the third set and neurospheres were maintained in normoxia as a control. At 24 h exposure to hypoxia, the cells in all sets were treated with temozolomide. One set of cells was then maintained in hypoxia; the other set was transferred to normoxia (Pre-H) while the third set was maintained in normoxia. The final viability of the cells was determined by AlamarBlue assay while GraphPad prism6 software was used to calculate IC_50_. N = 4. **c** Fold difference of temozolomide resistance in the 2D and 3D models in normoxia and hypoxia. *p* value is as indicated

To determine the role of the 3D tumour microenvironment in resistance to TMZ, GBM cells (U87, U251 and SNB19) were cultured in the 2D and 3D assays in normoxia and hypoxia. We found that cells cultured in the 3D assay were significantly more resistant to TMZ than those maintained as 2D in both normoxia and hypoxia with as high as fivefold in the U251 cells (Fig. 4a and c).

To further evaluate the role of different microenvironment in TMZ resistance, the U251 cells were used as model because they readily form neurospheres compared to the U87 and the SNB19 cells do not form neurospheres when they are first maintained as 2D. Two sets of U251 cells were exposed to hypoxia and a third set was maintained in normoxia as a control. The set maintained in normoxia was cultured as either 2D (monolayer) or neurosphere. After 24 h in hypoxia, cells in all sets were treated with temozolomide. From the two sets initially exposed to hypoxia, one set was immediately transferred to normoxia (24 h pre-exposure to hypoxia-Pre-H) (Fig. 4b). After 72 h of treatment with temozolomide, it was surprisingly found that exposure to hypoxia did not have any significant change to the resistance of temozolomide in the U251 cells (Fig. 4b). In addition, stem cell condition, i.e. the cells cultured as neurosphere, did not influence the resistance of cells to temozolomide (Fig. 4b).

To validate that the increase in resistance was an effect of the 3D environment, cells were first cultured as 3D, after 48 h, the cells were harvested from the 3D matrix and cultured as 2D and then treated with temozolomide. It was found that the IC_50_ values returned to baseline (not shown).

## Discussion

The realization that there is an obvious disconnect between in vitro culture and in vivo milieu has given researchers the reason to develop physiologically relevant in vitro assays that better reflect the tumour-microenvironment. The 2D monolayer culture system, though spontaneous and easily manipulated, has generated scientific concerns, as some drug agents that have been successfully classed as promising in this system have become clinically irrelevant, with only about 10% making it to the clinic leading to money and time wastage [7]. For instance, it has been shown that GSCs rely on kinases in the 2D substrates but not in a 3D environment [17]. Furthermore, many key players of the tumour-microenvironment are omitted in the 2D assays [18, 19]. As such, most conclusions from the 2D experiments do not take into account the influence of the tumour-microenvironment [20]. In this study, we described a novel glioblastoma 3D in vitro assay where hypoxia was incorporated to determine gene expression, cancer cell metabolism and chemoresistance.

We observed that the SNB19 cells formed neurospheres only when they were first cultured in the 3D assay. This may be as a result of cell reprograming as the tumour microenvironment can reprogram cells towards a stem-like phenotype [21, 22]. Furthermore, neurospheres formed from cells first cultured as 2D monolayers were bigger than those formed from cells generated from the 3D assays. Although, all neurospheres do not arise from stem cells [23], our observation could be related to the fact that true stem cells grow slowly than their differentiated progeny [24, 25].

It may seem that cells cultured as either 2D, 3D, or neurospheres are at different stages of lineage differentiation as seen by stem cell gene expression. While CD133 expression was upregulated in the neurosphere assay, OCT4 was upregulated in the 3D assay. Our result supports a system where multiple stem cell markers could be used to select stem cell population [26, 27].

Although, we did not use special markers, to immediately distinguish between stem cells and their transit amplifying progeny, it is crucial to evaluate which CD133 positive population constitute true stem cells population as the strengths and limitations of these systems (3D and neurosphere) need to be understood.

Metabolism plays a crucial role in GBM progression [28, 29] and it has been found that metabolic markers are upregulated in hypoxic tumour microenvironment [30, 31]. Our current result also indicates that there was an initial decrease in metabolism in the 3D-TGA compared to the cells cultured as 2D. This is consistent with the findings of Smith et al. [32] who used magnetic resonance spectroscopy to reveal differential metabolic profiles in 2D and 3D conditions. When cells are cultured in 3D, different zones of proliferation are introduced as a result of oxygen, nutrient and waste gradient. However, in the 2D assay, the rate of proliferation of cells is relatively uniform across the plate making growth of cells as 2D relatively faster than as 3D [33].

All our cells showed an initial lag in the 3D when compared to cells in the 2D, which is consistent with Kievit and colleagues who reported an initial lag of glioblastoma cells in the 3D chitosan-alginate scaffold [34].

Temozolomide (TMZ) has improved the prognosis of glioblastoma patients with its ability to cross the blood–brain barrier (BBB) and a bioavailability of 100% [35]. It has previously been observed that regions of hypoxia are prevalent in glioblastoma and that hypoxia selects for a highly chemoresistant phenotype in glioblastoma [36]. Most cells are resistant to chemotherapy in hypoxia [14].

However, when glioblastoma cells were cultured under a hypoxic environment in the 2D model, there was no significant difference in temozolomide resistance when compared with cells cultured in the same 2D model but under normoxic condition. Furthermore, when cells were cultured as neurospheres, which select for stem cell population, IC_50_ values were similar to those obtained in the 2D model. However, when the cells were cultured in the 3D model, we found that glioblastoma cells were significantly resistant to temozolomide and this was potentiated by hypoxia. It is important to highlight that hypoxia which was of no consequence in the 2D model played an important role in the 3D model implying that our 3D model may recapitulate in vivo temozolomide resistance and may allow for the study of components of the microenvironment that are involved in temozolomide resistance.

Cells cultured in the 3D model make contact with each other and with the matrix. The basement membrane extract used in this study is rich in collagen 4. In line with this, collagen 4 has been shown to promote the resistance of cells in vitro. Collagen 4 staining was also observed in vivo ovarian tumors were they correlated with tumor grade [37]. Laminin is another component of this 3D model. Interestingly, gene microarray analysis has shown that the α4 chain of laminin, which is a major blood vessel component, is overexpressed in human glial tumors [38]. While laminin-9 was associated with an astrocytoma of lower grades, high levels of laminin-8 were found in GBM and were associated with patient survival [39]. Moreover, brain tumors recurred faster in patients after standard therapy if laminin 8 is overexpressed and these patients had shorter survival time [40]. These results suggest that tumor cells may directly remodel their microenvironment to increase their survival in the presence of chemotherapeutic drugs and offers the opportunity to correlate basement membrane proteins with drug resistance in future experiments.

Thus, these finding implies that the resistance of GBM cells to temozolomide may not be adequately understood in isolation of components of the TME (in this case, hypoxia and components of the 3D model) because interaction of GBM cells with basement membrane proteins was enough to enhance drug resistance suggesting that stem cell populations may not be the only factor facilitating the resistance of glioblastoma to temozolomide. Indeed, downregulation of CD133 did not sensitise glioblastoma cells to temozolomide [14] but OCT4 expression is associated with tumour malignancy in astrocytic brain tumours [41] and indicates negative prognosis in Cervical Squamous Cell Carcinoma [42]. Moreover, knockdown of OCT4 increases the sensitivity to temozolomide in glioma-initiation cells [43] making it a target for the development of future therapeutic strategies [44]. Our 3D model reveals upregulation in OCT4 mRNA. These cells, along with those that co-express CD133 could be further exploited for future studies.

Overall, this study has demonstrated the possibility of maintaining and manipulating glioblastoma cells in vitro in a Cultrex-based 3D model. It was found that cells cultured in the 3D model behaved differently to their 2D counterparts, both morphologically and in response to chemotherapy. This model tended towards a highly-resistant and stem-like phenotype suggesting that it could be a highly-predictive surrogate model for in vitro high-throughput drug testing to understand and overcome temozolomide resistance in glioblastoma.

## Electronic supplementary material

Below is the link to the electronic supplementary material.

Supp. Fig. 1: U87 cells cultured as 2D, neurospheres, and 3D. Pictures were taken with a T9 Nikon Microscope. Scale bar = 100 μm. Magnification = ×10. (TIF 443 KB)

Supp. Fig. 2: Metabolic activity of cells in the 2D and 3D assays in normoxia and hypoxia: U87 cells were cultured in the 2D (grey) and 3D (black) assays. At day 0 of set up, baseline reading was taken with the Alamar Blue assay after the cells had settled and one set of the cells was maintained in normoxia (A) while the other set was transferred to hypoxia (B). The metabolic activity of the cells was monitored for 5 days. The graph was plotted relative to day 0. N = 1. (TIF 308 KB)
