# Supplementary figures and images for: A novel 3D in vitro model of glioblastoma reveals resistance to temozolomide which was potentiated by hypoxia
Source: J Neurooncol. 2019 Jan 29;142(2):231–40. doi: 10.1007/s11060-019-03107-0 (PMC6449313; doi:10.1007/s11060-019-03107-0)

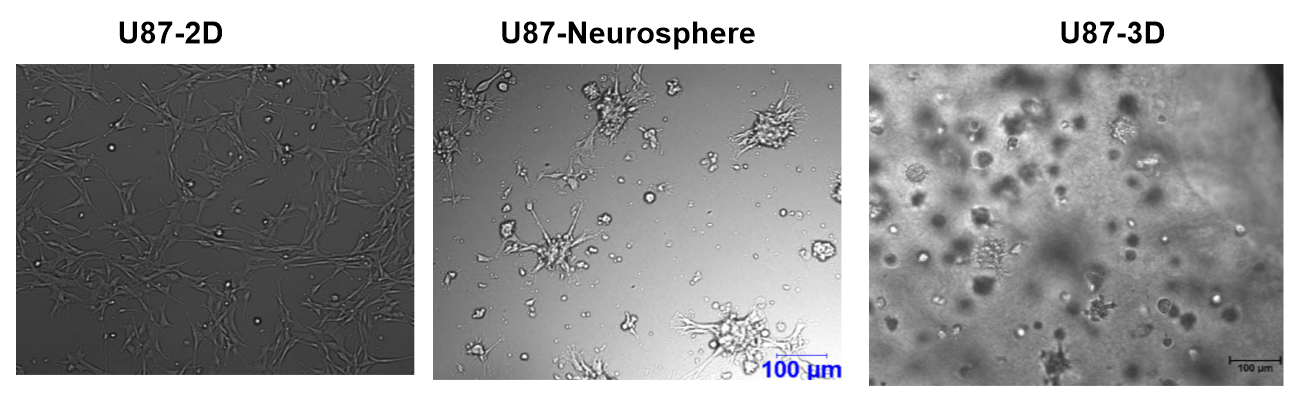

Supplement: Supplementary file 1 — Supp. Fig. 1: U87 cells cultured as 2D, neurospheres, and 3D. Pictures were taken with a T9 Nikon Microscope. Scale bar = 100 μm. Magnification = ×10. (TIF 443 KB) [file 11060_2019_3107_MOESM1_ESM.tif]

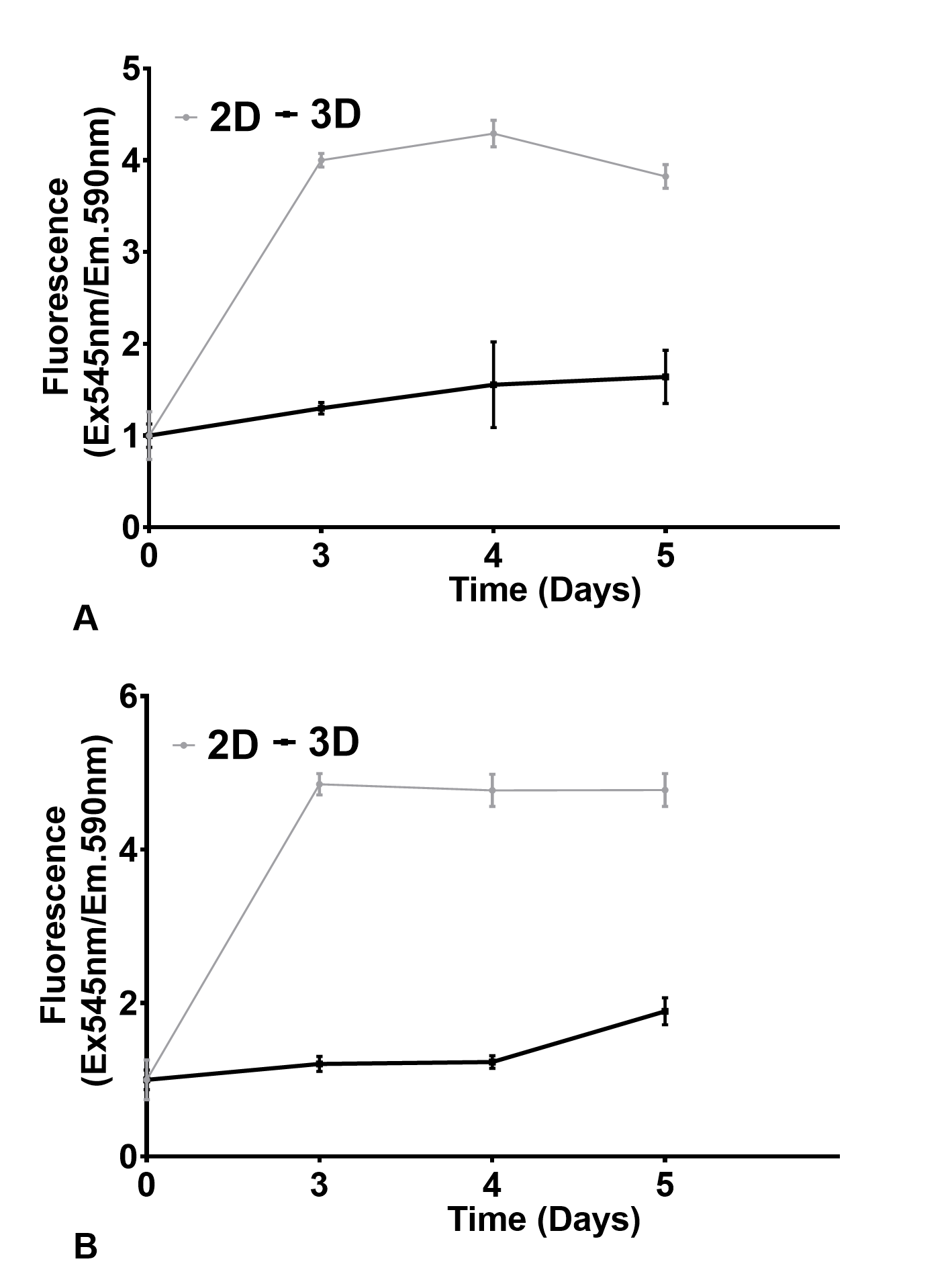

Supplement: Supplementary file 2 — Supp. Fig. 2: Metabolic activity of cells in the 2D and 3D assays in normoxia and hypoxia: U87 cells were cultured in the 2D (grey) and 3D (black) assays. At day 0 of set up, baseline reading was taken with the Alamar Blue assay after the cells had settled and one set of the cells was maintained in normoxia (A) while the other set was transferred to hypoxia (B). The metabolic activity of the cells was monitored for 5 days. The graph was plotted relative to day 0. N = 1. (TIF 308 KB) [file 11060_2019_3107_MOESM2_ESM.tif]
